# Within- and Trans-Generational Effects of Variation in Dietary Macronutrient Content on Life-History Traits in the Moth *Plodia interpunctella*

**DOI:** 10.1371/journal.pone.0168869

**Published:** 2016-12-29

**Authors:** Joanne E. Littlefair, Robert J. Knell

**Affiliations:** School of Biological and Chemical Sciences, Queen Mary University of London, London, United Kingdom; USDA Agricultural Research Service, UNITED STATES

## Abstract

It is increasingly clear that parental environment can play an important role in determining offspring phenotype. These “transgenerational effects” have been linked to many different components of the environment, including toxin exposure, infection with pathogens and parasites, temperature and food quality. In this study, we focus on the latter, asking how variation in the quantity and quality of nutrition affects future generations. Previous studies have shown that artificial diets are a useful tool to examine the within-generation effects of variation in macronutrient content on life history traits, and could therefore be applied to investigations of the transgenerational effects of parental diet. Synthetic diets varying in total macronutrient content and protein: carbohydrate ratios were used to examine both within- and trans-generational effects on life history traits in a generalist stored product pest, the Indian meal moth *Plodia interpunctella*. The macronutrient composition of the diet was important for shaping within-generation life history traits, including pupal weight, adult weight, and phenoloxidase activity, and had indirect effects via maternal weight on fecundity. Despite these clear within-generation effects on the biology of *P*. *interpunctella*, diet composition had no transgenerational effects on the life history traits of offspring. *P*. *interpunctella* mothers were able to maintain their offspring quality, possibly at the expense of their own somatic condition, despite high variation in dietary macronutrient composition. This has important implications for the plastic biology of this successful generalist pest.

## Introduction

For all animals, dietary nutritional resources are essential for the provisioning of the soma, as well as for providing energy and nutrients that will be used for reproduction. Trade-offs as a result of variation in the availability of these resources are a powerful shaper of phenotype, affecting a range of traits from an organism’s growth and survival to costly signals of quality such as sexual ornamentation [1–4]. In addition to allocation to parental traits, nutritional resources can also contribute to the provisioning of eggs and resultant offspring, so offspring condition might therefore be constrained by any deficits in parental nutrition [5–7]. Dietary resources vary in quality as well as quantity, and the relative and absolute amounts of macronutrients (e.g. protein, carbohydrates, lipid) within the food source are one such variable. While nutritional ecologists have mapped the within-generational effects of macronutrients on life history traits and the immune system [8–11], to date there has been very little research on the transgenerational consequences of specific macronutrients in the parental diet.

These limitations notwithstanding, researchers using oligidic diets (containing complex organic ingredients of a poorly-defined nature, such as bran, wheat germ etc.) have experimentally manipulated parental resource allocation to offspring, demonstrating effects on the growth rate of immature stages, weight of the imago, mortality and ultimately the reproductive output of the offspring [12–15]. Parental diet has also been shown to affect offspring immune function, which decreases when both mothers and fathers are nutritionally stressed [16]. Although generally detrimental to offspring phenotype, in some cases a poor parental diet can alter per-offspring investment, creating a “thrifty” offspring phenotype which is adaptive in poor conditions [17–19]. For example, one study showed that nutritionally stressed *Daphnia magna* produced offspring that were more resistant to the parasite *Pasteuria ramosa* [20]. Subsequent research demonstrated that this was a result of decreased rates of algae filtration from the water, which reduces their exposure to the parasite [21].

The within-generation consequences of specific macronutrients on life history traits have been well defined. For example, it is well established that dietary protein is essential for reproduction and growth [22–24]. Increased dietary protein also raises the levels of constitutive immune defences in the haemolymph, and can provide additional nitrogen resources for the production of immune cells and enzymes [25,26]. Invertebrate mothers provision their eggs with yolk proteins, and stable isotope studies have shown that the amino acids in these proteins are derived from the larval maternal diet [27,28]. Carbohydrates are a major macronutrient necessary for processes with high energy requirements, such as movement, somatic maintenance and growth [29]. In some cases, carbohydrates are also implicated in defence against pathogens [30], even though the immune system is generally thought to be protein-limited. Insect lipid requirements include free and bound fatty acids, steroids, acylglycerols, and phospholipids [31]. Specific fatty acids are critical for pupation and ecdysis, and without them metamorphosis will fail. Fatty acids are also a rich source of energy (greater than protein sources), and their accumulation is particularly important in organisms which have non-feeding adult stages, such as some Lepidoptera species [32].

When we examine the within- and trans-generational consequences of diet, we must define what is meant by variation in the quality or quantity of nutrition. Researchers often rely on starvation or dilutions of oligidic diets to create experimental variation in nutritional quality [13,16,33,34]. If oligidic diets are used, this means that the quantity of different macronutrients in the diet (e.g. carbohydrates, protein, lipid) is unknown, and the effects of the different types of macronutrients are confounded by the total amount of calories within the diet [5,6]. Finally, this type of experimental design is often limited by the inclusion of just two levels within the explanatory variable (e.g. starved and non-starved, high nutrient and low nutrient), which means that we cannot see a full range of responses in investment.

Artificial diets have been used to great effect in within-generation studies manipulating the macronutrient content of diet [8,22,35,36], and could therefore be a useful tool for examining the transgenerational consequences of parental diet. They remove the use of yeast as a protein source in diets for Lepidoptera, which could be providing stimulation to the immune system by stimulating the immune response via pathogen-associated molecular patterns (PAMPs) on the cell surface or even by acting as an opportunistic pathogen itself. Finally, isocaloric diets can be created with varying proportions of protein and carbohydrates, allowing us to separate the confounding effects of altering calories and macronutrient intake [37].

In this study we performed experiments to characterise the effects of variation in both the total quantity of macronutrients (by diluting isocaloric macronutrients with non-digestible cellulose), and the ratio of protein to carbohydrates within each of the three levels of macronutrient dilution, using artificial diets developed in our laboratory [38]. We characterised the within-generation effects of the six diets on the condition of the Indian meal moth *Plodia interpunctella* in both males and females, measuring eclosion and pupation weight, fecundity and constitutive immune activity (haemocyte counts and phenoloxidase activity). Haemocytes are the effector cells of the invertebrate immune system. Their density is tightly correlated with the ability to resist a bacterial or viral attack [39], whereupon they produce cytotoxic molecules or phagocytose pathogens [40]. Part of the humoral immune system, phenoloxidase (PO) is a key enzyme in wound repair, parasite encapsulation and cuticle defence [41,42], which are essential processes to limit the spread of pathogens. We additionally examined maternal effects resulting from the six larval diets on the phenotype of offspring. Only daughters were examined in the F_1_ generation of this experiment due to logistical constraints. The experiment revealed that there are within-generational effects of dietary protein and carbohydrates on life history traits, but no transgenerational effects on the phenotype of offspring.

## Methods

### Creating the artificial diets

Six artificial diets based on the diet described in [38] were created containing three different levels of non-nutritive bulking agent (30%, 50% and 70% cellulose), with the remainder consisting of either two-thirds protein and one-third carbohydrates or one-third protein and two-thirds carbohydrates. In addition, 1% cholesterol, 1% Vanderzant vitamin mixture, 1% Wesson salts, 4% glycerol and 4% linseed oil were added to each diet.

### Experiment 1: Within-generation effects of dietary macronutrients

Approximately 200 adults were taken from the stock population of *Plodia interpunctella* and allowed to mate. Their eggs were collected after 24 hours and placed on laboratory food consisting of 10:1:1 wheat bran: brewers’ yeast: glycerol. After 15–16 days the resulting larvae reached the third instar and were weighed and placed in individual 55mm width Petri dishes with a 0.8g block of one of the six artificial diets. Larvae were fed on the artificial diet from third instar onwards because smaller larvae sometimes drowned in the slightly wet diet. The artificial diet was replaced with fresh diet every two days to prevent desiccation. Larvae were maintained in a temperature controlled room at 27°C on a 12:12 light/dark cycle for the duration of the experiment.

Half of the larvae were allowed to pupate and then eclose (n = 333). Their pupal weight was recorded, and on the day the adults eclosed, they were sacrificed by freezing and weighed to obtain adult weight (Sartorius BP2215 balance, d = 0.1mg). The other half of the larvae were assayed for two constitutive components of larval immunity (see details in immune assays section; haemocyte counts: n = 393, phenoloxidase activity: n = 375). The experiment was carried out as four experimental blocks distributed over a ten month period (April 2013 –January 2014).

### Experiment 2: Transgenerational effects of macronutrient ratios in maternal diets

Approximately 200 adults from the stock population were allowed to mate and the eggs reared until third instar on laboratory diet. At third instar, larvae were transferred onto the six artificial diets as above. The diet was changed every two days until the larvae began to pupate. Males were discarded from the experiment at fifth instar, when the testes become visible on the dorsal side of the larva. Female moths were weighed as pupae and were mated with a freshly eclosed virgin male from the stock population once they had eclosed. After 24 hours, the male was removed, and the number of eggs laid by the female was recorded daily until she died. Eggs from the first 72 hours of laying were reared on the bran-based laboratory food (described above) and formed the offspring generation of the experiment. Offspring were reared until fifth instar, when they were separated into three treatment groups within each family. Group 1 was reared until eclosion, when they were sacrificed by freezing and weighed to obtain adult weight at eclosion (n = 266). Group 2 was allowed to eclose and were then monitored daily for survival until they died to obtain longevity data (n = 159). Group 3 was sacrificed when they reached the wandering phase of fifth instar, in order to assay immune traits (haemocyte counts: n = 342, phenoloxidase activity: n = 325). The maternal effects experiment was carried out as seven experimental blocks distributed over a ten month period (June 2013—March 2014).

### Immunity assays

Larvae were allowed to grow until they reached the wandering phase of the fifth instar. A haemolymph sample was taken by pricking the larva with a sterile needle (BD Microlance, 0.5mm tip, 25mm length) and allowing the haemolymph to pool out on to Parafilm. 1μl haemolymph was transferred into 10ul chilled sterile PBS in a 0.2ml microtube, and stored at -80°C for the phenoloxidase assay. 1μl was transferred into a 0.2ml microtube containing 3μl chilled EDTA buffer in PBS and 4μl glycerol, which allows the sample to be frozen without disrupting the haemocytes [2]. All samples were collected using sterile technique, and were collected at the same time of day to avoid possible confounding circadian variations in haemocyte numbers.

Circulating haemocyte levels were assayed by pipetting 4μl of sample on two sides of a haemocytometer (Neubauer improved brightline haemocytometer, 0.1mm depth, 0.0025mm^2^, Assistent, Germany) and counting the haemocytes in four corner squares and one central square of the grid on each side of the haemocytometer, so that 10 measurements for each individual were made in total.

Phenoloxidase assays were performed by vortexing and centrifuging each sample for 15 seconds using a Jencons-PLS mini bench centrifuge and transferring them to flat-bottomed 96-well plates. 100μl of 4mg/ml L-DOPA was added to each sample in the plate. They were incubated in an Ascent v2.6 plate reader (Thermo labsystems) at 30°C for one hour and absorbance was measured every 15 seconds at 492nm. Each plate included two blank wells containing 9μl PBS on each plate, and blank values were subtracted from the experimental wells on each plate at each time point. Vmax (the slope of the reaction curve during the linear phase) of the reaction was calculated [43] using in-house R scripts.

### Statistical analysis

All analyses were conducted using R version 3.0.1 [44].

#### Experiment 1 analysis (within-generation)

The effects of diet on pupal weight, adult weight, haemocyte counts and phenoloxidase activity were analysed with linear regressions, unless otherwise specified. The total amount of macronutrients, ratio of dietary protein:carbohydrates, and their interaction were included as initial fixed factors, so as to assess the effect of macronutrient content and ratio of macronutrients nested within it. Model reduction was performed using deletion tests to assess which terms contributed significantly to model fit, and removing non-significant terms to create the minimum adequate model [45]. For the immunity measures (haemocyte count and PO activity), larval weight at the time of sacrifice was also included as a covariate. Phenoloxidase vmax was reciprocal-transformed to improve model fit. Fecundity was analysed as a mixed effects model with the nlme package in R [46], using experimental block as a random effect and maternal weight and adult lifespan as additional fixed effect covariates.

#### Experiment 2 analysis (maternal effects)

Mixed effects models were used for all analyses unless otherwise stated. The total amount of macronutrients in the maternal diet, ratio of dietary protein: carbohydrates, and their interaction were included as initial fixed factors. Family identity was nested within experimental block and included as a random effect. Model reduction was performed using likelihood ratio tests to compare models fitted using maximum likelihood, and fixed effects terms were discarded if there was no significant difference between models with and without them [47]. Minimum adequate models were refitted using REML to obtain final estimates and standard errors. Offspring phenoloxidase vmax was square-root transformed after examination of model diagnostic plots to reduce heteroscedasticity. Longevity data (adult lifespan and larval development time) were analysed with Cox proportional hazards models with family nested within block included as random effects using the R package “coxme” [48], and model reduction was performed with likelihood ratio tests, as above.

## Results

Full tables of statistical tests can be found in the supporting information (S1, S2 and S3 Tables).

### Experiment 1 (within-generation)

#### Pupal weight

**Fig 1:**
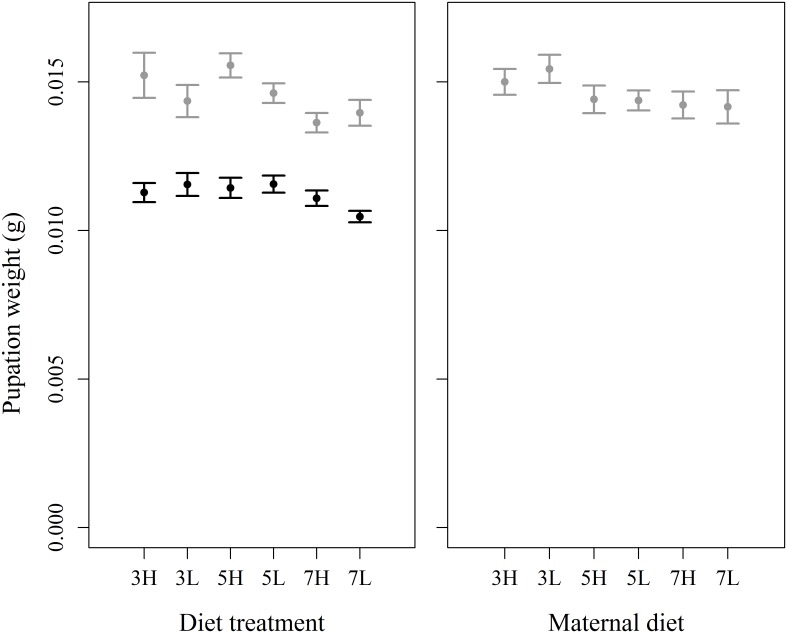
Effects of within-generation and transgenerational variation in dietary macronutrients on pupation weight. There was a significant effect of total macronutrient content (protein and carbohydrates combined) and a separate main effect of sex in the within-generation experiment (left panel), but no transgenerational effects from maternal diet (right panel). Black bars: males, grey bars: females; X axis labels refer to diet composition, e.g. 3H = 30% cellulose, high protein, 5L = 50% cellulose, low protein; error bars are 95% confidence intervals.

There was a significant effect of dietary macronutrient (protein and carbohydrates) content on pupation weight (Fig 1). Pupae weighed less when they consumed diets with the lowest amount of dietary macronutrients (30% cellulose = 0.0126g, 50% cellulose = 0.0130g, 70% cellulose = 0.0118g, F_2, 328_ = 7.59, P = 0.0006). There was no effect of P:C ratio (F_1, 327_ = 1.62, P = 0.204), or the interaction between P:C ratio and total amount of dietary macronutrient content (F_2, 321_ = 0.085, P = 0.918). There was a separate significant main effect of sex, whereby females were heavier than males (females = 0.0145g, males = 0.0112g, F_1, 328_ = 225, P < 0.001). Experimental block was also significant (F_3, 328_ = 3.67, P = 0.0126: see S1 Table for details of the block effects here and in subsequent analyses).

#### Adult weight

**Fig 2:**
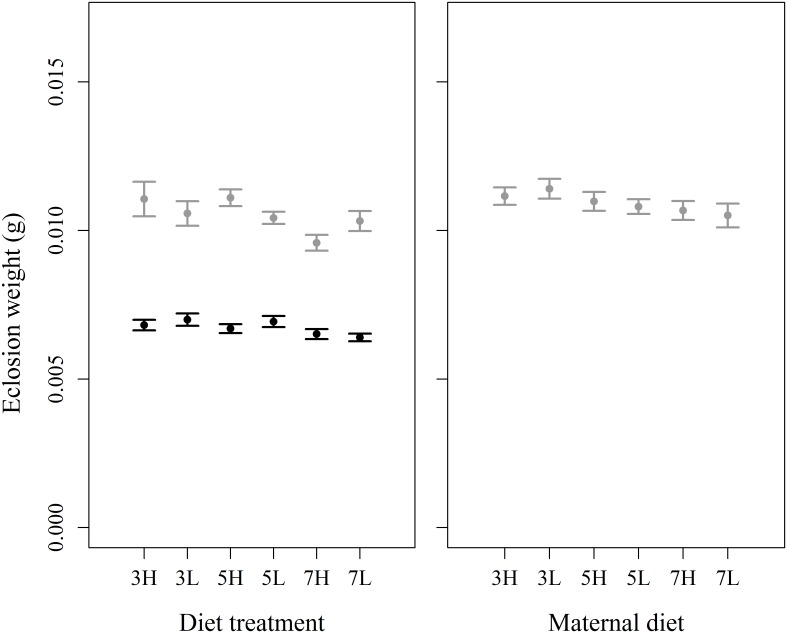
Effects of within-generation and transgenerational variation in dietary macronutrients on adult weight. A three way interaction between total macronutrient content, P:C ratio, and sex shaped the response of adult weight to diet composition in the within-generation experiment (left panel). There were no transgenerational effects of maternal diet composition (right panel). Black bars: males, grey bars: females; X axis labels refer to diet composition, e.g. 3H = 30% cellulose, high protein, 5L = 50% cellulose, low protein; error bars are 95% confidence intervals.

There was a significant 3-way interaction between the total amount of macronutrient content, P:C ratio and sex on adult weight at eclosion (F_2, 320_ = 3.15, P = 0.044). From Fig 2 and also examination of the Wald P-values in the model summary table, it can be seen that the strongest difference is between the sexes, whereby females are heavier than males (females = 0.0105g, males = 0.0067g). Males appear to be more resilient to variation in dietary macronutrients, whereas female adult weight varies more in response to the different diet treatments (Fig 2). There was also a significant effect of experimental block (F_3,320_ = 5.62, P = 0.0009).

#### Immunity measures

**Fig 3:**
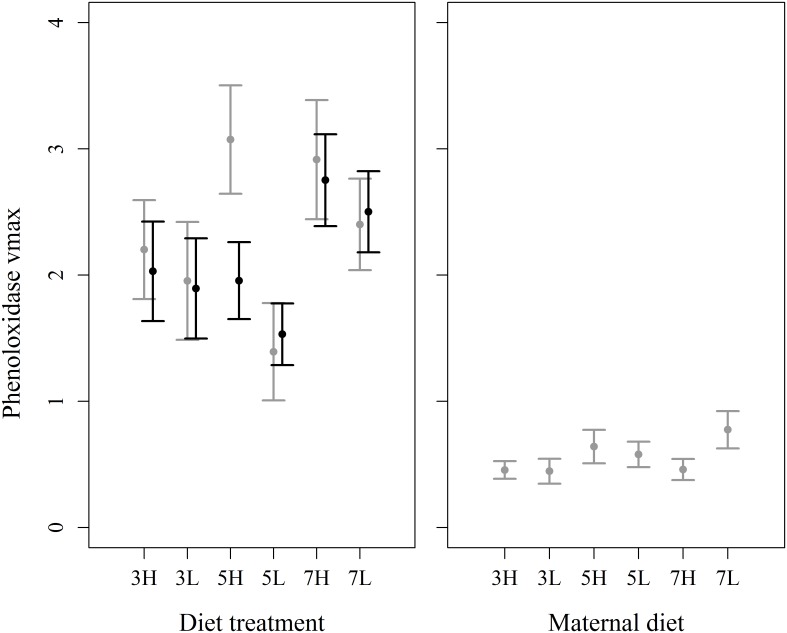
Effects of within-generation and transgenerational variation in dietary macronutrients on phenoloxidase vmax. The interaction between total macronutrient content (protein and carbohydrates combined) and sex shaped the response of phenoloxidase activity to diet composition in the within-generation experiment (left panel). There were, however, no transgenerational effects of maternal diet composition (right panel). Black bars: males, grey bars: females; X axis labels refer to diet composition, e.g. 3H = 30% cellulose, high protein, 5L = 50% cellulose, low protein; error bars are 95% confidence intervals.

The response variable of phenoloxidase vmax was reciprocal transformed following inspection of diagnostic plots in order to meet the assumptions of normal errors. Phenoloxidase vmax was influenced by a significant interaction between macronutrient content and sex (F_2, 378_ = 3.22, P = 0.041). Both sexes had higher levels of phenoloxidase activity when consuming the diet with the lowest amount of macronutrient content, but males and females responded differently to diets containing 50% cellulose (Fig 3). A close examination of the raw data revealed that this pattern was probably driven by ~6 females with higher than expected PO activity in this one treatment. Given this and the weak nature of the interaction, we are not confident that this increased activity in females given 50% cellulose is not a type 1 error. There were no effects produced by variation in P:C ratio (F_1, 377_ = 1.20, P = 0.274) or the interaction between macronutrient content and P:C ratio (F_2, 375_ = 0.640, P = 0.528). There were separate significant main effects of larval weight at the time of sacrifice (F_1, 378_ = 19.6, P < 0.001), and experimental block (F_3, 378_ = 21.1, P < 0.001).

**Fig 4:**
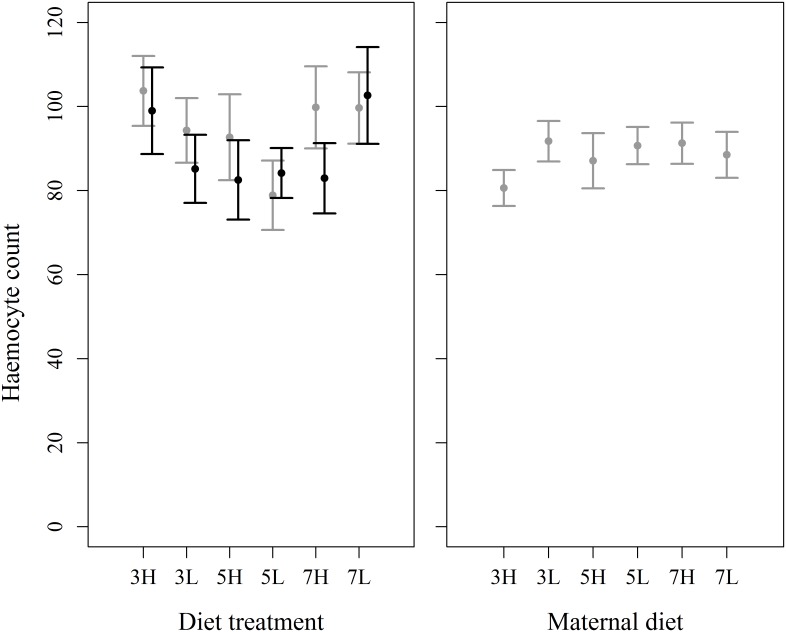
Effects of within-generation and transgenerational variation in dietary macronutrients on haemocyte counts. There were no within-generation or transgenerational effects of dietary composition on haemocyte count. Left panel: within-generation experiment, right panel: transgenerational experiment; black bars: males, grey bars: females. X axis labels refer to diet composition, e.g. 3H = 30% cellulose, high protein, 5L = 50% cellulose, low protein; error bars are 95% confidence intervals.

Total haemocyte count was not affected by any dietary components (Fig 4), including total macronutrient content (F_2, 380_ = 2.31, P = 0.101), P:C ratio (F_1, 378_ = 0.490, P = 0.485) or the interaction between them (F_2, 375_ = 1.63, P = 0.198). There were no differences between the sexes (F_1, 379_ = 1.44, P = 0.231), but there was a significant effect of experimental block (F_3, 382_ = 12.4, P < 0.001) on the total haemocyte count.

#### Fecundity

**Fig 5:**
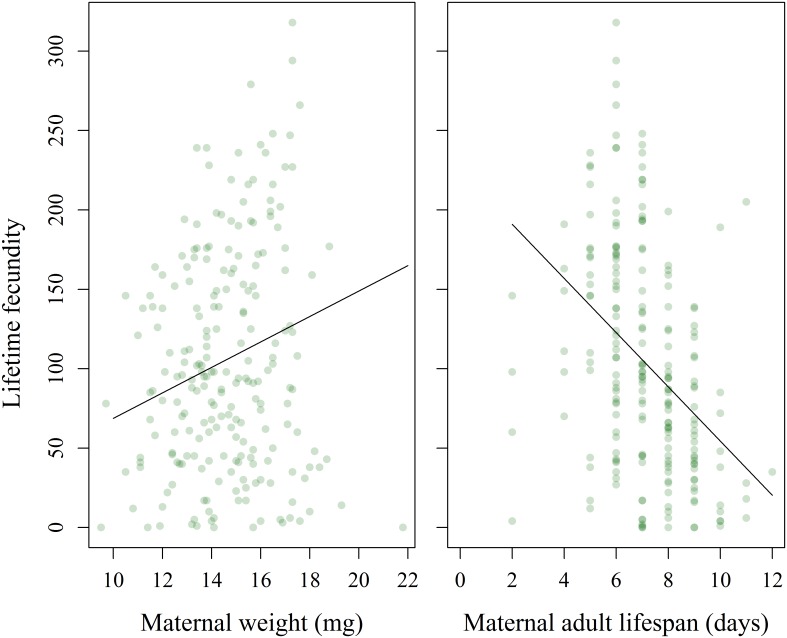
Effects of maternal weight and longevity on maternal fecundity. There was a positive relationship between maternal pupal weight and fecundity (left panel, slope = 8.01) and a negative relationship between maternal longevity and fecundity (right panel, slope = -17.065). Conditional r^2^ for model = 0.207, based on [49] and calculated with piecewiseSEM [50].

Fecundity was significantly affected by maternal weight, with heavier pupae producing more eggs after eclosion (Fig 5, likelihood ratio = 4.72, P = 0.031). We found a trade-off between fecundity and maternal longevity, with mothers dying a day earlier for every additional 17.5 eggs they produced (Fig 5, likelihood ratio = 42.4, P < 0.001). There were no direct effects of variation in diet quality on fecundity, including total macronutrient content (likelihood ratio = 0.275, P = 0.872), P:C ratio (likelihood ratio = 1.15, P = 0.283), or the interaction between them (likelihood ratio = 1.90, P = 0.387). However, it is likely that diet indirectly affected fecundity through maternal weight, because there were significant effects of macronutrient composition on maternal weight (see sections on pupation and adult weight).

### Experiment 2 (trans-generational effects in female offspring)

#### Pupal weight

There were no significant effects of variation in maternal diet on daughters’ pupation weight (Fig 1), including total macronutrient content (χ^2^ = 4.22, P = 0.121), dietary P:C ratio (χ^2^ = 0.013, P = 0.910), or the interaction between them (χ^2^ = 0.603, P = 0.740). Maternal pupal weight also did not have a significant effect on pupation weight (χ^2^ = 0.0227, P = 0.880).

#### Adult weight

Maternal diet did not influence daughters’ weight upon eclosion (Fig 2). There were no significant effects of dietary macronutrient content (χ^2^ = 3.81, P = 0.149), P:C ratio (χ^2^ = 0.038, P = 0.844), or the interaction between them (χ^2^ = 0.717, P = 0.699). There was also no effect of maternal pupal weight on daughters’ adult weight (χ^2^ = 0.025, P = 0.874).

#### Immunity measures

The response variable of phenoloxidase vmax was square-root transformed to improve the appearance of the model validation plots, and reduce heteroscedasticity. Heavier larvae had lower levels of phenoloxidase activity (χ^2^ = 12.1, P = 0.0005). There were, however, no effects of variation in the maternal diet on offspring phenoloxidase activity (Fig 3), including the total macronutrient content (χ^2^ = 1.04, P = 0.595), P:C ratio (χ^2^ = 0.245, P = 0.621), or the interaction between them (χ^2^ = 4.13, P = 0.127).

Similarly, total haemocyte counts were not influenced by maternal dietary components (Fig 4), including total macronutrient content (likelihood ratio = 0.372, P = 0.830), P:C ratio (likelihood ratio = 1.99, P = 0.159), or the interaction between them (likelihood ratio = 1.10, P 0.576). However, heavier larvae had lower haemocyte counts (likelihood ratio = 7.04, P = 0.0086).

#### Larval development time

Neither total macronutrient content (χ^2^ = 2.73, P = 0.256), P:C ratio (χ^2^ = 1.95, P = 0.163) or the interaction between them (χ^2^ = 1.48, P = 0.478) in maternal diet influenced the number of days from egg to pupation.

#### Adult lifespan

Neither total macronutrient content (χ^2^ = 2.17, P = 0.338), P:C ratio (χ^2^ = 0.245, P = 0.621) or the interaction between them (χ^2^ = 1.15, P = 0.562) in the maternal diet influenced the number of days from eclosion to death. There was a separate main effect of pupal weight on adult lifespan, where heavier pupae had longer adult lifespans (χ^2^ = 50.1, P < 0.001).

## Discussion

In this experiment we manipulated the total quantity of dietary macronutrients as well as protein and carbohydrate content in order to study the within-generation and transgenerational consequences of macronutrient variation. Previously, non-chemically defined diets have mainly been used to examine transgenerational effects in invertebrates, which do not allow tests of the relative importance of each macronutrient to resource allocation. In this study we found that life history traits in *Plodia interpunctella* show within-generation variation in response to dietary macronutrient content, but they appear to be robust to any transgenerational effects.

Increasing dietary protein and carbohydrates had positive effects on pupation weight and adult weight in the within-generation experiments. Previous studies have shown that the growth of the soma is limited by protein consumption [22,25,51], as protein contributes towards muscle and tissue development. This has important consequences for capital breeding insects, as body mass is often linked with fecundity, flight, or the production of sexual ornaments [52–54]. Production of haemocytes is often found to be reliant on protein intake, due to their high protein content [25,55], but this was not the case in our experiment: there were no within- or transgenerational effects of diet on haemocyte count. The response of phenoloxidase to macronutrient intake seems to be variable, as other studies [11,25], have found that there is no relationship between the two. Instead, we found a negative relationship between total dietary macronutrient content and phenoloxidase activity. Further effects of diet on phenoloxidase levels can be seen from the differences in PO levels between the parents and offspring. We suggest that the lower levels in the offspring are due to the differences in bran-based and artificial agar-based diets. The lower phenoloxidase levels on the bran-based diet are consistent with the results from previous experiments on this animal [16]. Finally, the relationship between protein intake and immune traits is likely to become more pronounced when an immune challenge is initiated, as in [55], which we did not investigate here.

Macronutrient intake during the larval development stage shaped post-pupation traits across the metamorphic boundary. We found that larvae consuming high levels of dietary protein grew to heavier adult weights. Using the same artificial diets, we have also shown in another study that decreased dietary protein content in the larval stage lengthens lifespan post-eclosion (i.e. dietary restriction after metamorphosis) [38]. *P*. *interpunctella* do not feed as adults, and therefore the only resources available during adulthood are those consumed in the larval stage and allocated to adult traits during pupation. Studies in hemimetabolous species, or where holometabolous insects feed in both developmental stages, often show quite different trait responses to feeding or may display compensation through increased food uptake for deprivation in one developmental stage. For example, *Telostylinus angusticollis* displays lifespan extension with increased larval dietary protein content, but lifespan restriction when they feed on protein as adults [56]. This has interesting evolutionary implications linking larval feeding to the development of adult traits which are then linked to reproductive success and Darwinian fitness.

No transgenerational effects of dietary macronutrients consumed by parents were found in this study. Life history and immunity traits of *P*. *interpunctella* daughters were robust to deficiencies in dietary macronutrient composition which adversely affected their mothers. This is consistent with a meta-analysis of 58 transgenerational effects studies suggesting that the effect sizes produced by the mother’s environment are modest [57]. Previous experiments on invertebrates have found that when offspring traits are shaped by maternal effects [58,59], this often occurs via changes in egg number, size or composition. In our study, mothers did not alter their fecundity directly in response to variation in dietary composition. *P*. *interpunctella* mothers were instead able to maintain offspring numbers and condition, seemingly at the expense of their own somatic condition, in the face of adverse diet treatments, although there was an indirect effect of diet on fecundity mediated by maternal weight. Alternatively, catch-up growth by offspring in the early instars may be masking any effects from a poor maternal diet, as offspring all received the same nutritionally rich bran-based diet. However, the meta-analysis of transgenerational effects shows that maternal effects are modest even when the offspring’s environment is poor [57].

Other experiments have shown that the larval stages of invertebrates compensate for poor nutritional conditions by pre-or post-ingestive regulation of food intake. Pre-ingestive regulatory mechanisms in other successful generalist insects include the preferential consumption of depleted macronutrients, or phenotypic plasticity to increase food intake in poor conditions [60,61]. For example, under conditions of nutritional stress, *Daphnia* feeding screens enlarged up to 83% in order to increase the filter feeding rate [62]. Post-ingestively, the gastro-intestinal tract has been shown to enlarge in various arthropod species to increase the amount of food that can be processed, and to retain food within the gut for a longer time period [63–65]. Other mechanisms of post-ingestive regulation also include the egestion of excessive nutrients [61,66], and the conversion of excess carbohydrates to lipids for storage as energy reserves [66]. While nutrient regulation might be possible in either mothers or offspring in our study, mothers cannot be entirely compensating for their nutritional intake because of the significant within-generation effects on phenotype.

The lack of maternal effects found in this study must be reconciled with previous studies in *P*. *interpunctella* which found evidence for transgenerational effects of parental diet on the immune system [16]. In the study by Triggs and Knell (2012), oligidic diets contained dilutions of yeast to create variation in the amounts of dietary protein and vitamins. Yeast is a commensal but also opportunist pathogen. It is possible that the detection of yeast PAMPs explains the upregulated levels of constitutive immunity and evidence for parental effects in the immune system in [16], which were not found in our study.

Transgenerational effects have consequences for both the fitness of individuals and the dynamics of entire populations. Resource allocation-based maternal effects have interesting implications for parent-offspring conflict, which will occur when parents and offspring have different optimal strategies for allocation. For example, if a mother invests resources to impose her own optimal allocation between her present and future offspring, conflict could exist between them because individuals are selected to maximise their own fitness and not that of the parent [67]. If this is detrimental to the fitness of offspring, selection will favour reduced sensitivity to manipulative parental signals. The evolutionary dynamics of conflict will change depending on whether manipulation is expensive or cheap (for example, the transfer of information through hormones or epigenetic marks is not considered expensive, whereas the transfer of resources is) [67]. The quality of information transfer and extent of parental effects is uncertain in taxa which do not have intimate and prolonged contact between the generations through the shared phenotype of the mammalian placenta. Although the eggs of invertebrates are a shared phenotype of mother and offspring, it remains to be seen whether the development window allows for adequate time for information transfer about the mother’s external environment before the eggs become chorionated [13,68]. Other insects have obvious control over the environment of offspring, such as the telescopic generations of aphids, where in effect offspring environment is controlled by the mother [58]. Parental effects also act as a proximal source of time-lagged effects on population dynamics, especially if they affect fecundity in adult offspring (Rossiter 1996). The apparently robust nature of *P*. *interpunctella* to detrimental conditions experienced by previous generations may have important implications for their pest biology and ability to infest food production in large numbers [69].

## Supporting Information

S1 Table Full table of statistics for within-generation results. The within-generation effects of dietary macronutrient content on life history traits including pupation weight, eclosion weight, phenoloxidase activity and haemocyte count.
(PDF)

S2 Table Full table of statistics for maternal fecundity. Effects of dietary macronutrient composition on fecundity.
(PDF)

S3 Table Full table of statistics for transgenerational effects. Transgenerational effects of maternal dietary macronutrient composition on offspring life history traits.
(PDF)

S1 File Supporting information—Compressed/ZIP file archive. The raw data for within-generational experiments (files beginning “within_gen”), transgenerational experiments (files beginning “trans_gen”), and patterns of egg-laying (“fecundity_data”).
(ZIP)
