# Supplementary material for: Within- and Trans-Generational Effects of Variation in Dietary Macronutrient Content on Life-History Traits in the Moth Plodia interpunctella
Source: PLoS One. 2016 Dec 29;11(12):e0168869. doi: 10.1371/journal.pone.0168869 (PMC5199116; doi:10.1371/journal.pone.0168869)
Supplement: S1 Table — The within-generation effects of dietary macronutrient content on life history traits including pupation weight, eclosion weight, phenoloxidase activity and haemocyte count. (PDF) [file pone.0168869.s001.pdf]

Supporting information for "Within- and trans-generational effects of variation in dietary macronutrient content on life-history traits in the moth *Plodia interpunctella*"

Joanne E. Littlefair, Robert J. Knell

S1 Table: Full table of statistics for within-generation results. The within-generation effects of dietary macronutrient content on life history traits including pupation weight, eclosion weight, phenoloxidase activity and haemocyte count.

Single generation	Pupation weight (g)	Eclosion weight (g)	Phenoloxidase vmax (reciprocal transformation)	Total haemocyte count
Intercept	0.0146 ± 0.0003	0.110 ± 0.00032	0.130 ± 0.107	110 ± 4.48
3 way Interaction between nutrient content, P:C ratio and sex	F 2.10 (2, 319)<br>P 0.124	B 0.0002 ± 0.00069<br>C -0.0014 ± 0.00069<br>**F 3.15 (2, 320)**<br>**P 0.044**	F 1.06 (2, 372)<br>P 0.346	F 0.265<br>P 0.768 (2,370)
Nutrient content:sex interaction	F 0.62 (2, 323)<br>P 0.538	B -0.00010 ± 0.00050<br>C 0.0011 ± 0.00049	0.0420 ± 0.0657<br>-0.119 ± 0.0637<br>**F 3.22 (2, 378)**<br>**P 0.041**	F 0.191 (2, 372)<br>P 0.826
P:C: sex interaction	F 0.612 (1, 325)<br>P 0.434	0.00059 ± 0.00050	F 0.038 (1, 374)<br>P 0.846	F 1.63 (1,377)<br>P 0.203
Nutrient content: P:C ratio interaction	F 0.085 (2, 321)<br>P 0.918	B -0.00014 ± 0.00054<br>C 0.0011 ± 0.00056	F 0.640 (2, 375)<br>P 0.528	F 1.63 (2,375)<br>P 0.198
Macronutrient content	0.00017 ± 0.00026<br>-0.00075 ± 0.00025<br>**F 7.59 (2, 328)**<br>**P 0.0006**	-0.000012 ± 0.00040<br>-0.0014 ± 0.00040	-0.0923 ± 0.049<br>-0.0203 ± 0.044	F 2.31 (2, 380)<br>P 0.101
P:C ratio (low protein, high carbohydrate)	F 1.62 (1, 327)<br>P 0.204	-0.00047 ± 0.00040	F 1.20 (1, 377)<br>P 0.274	F 0.490 (1, 378)<br>P 0.485
Sex (males)	-0.0032 ± 0.00022<br>**F 225 (1, 328)**<br>**P < 0.001**	-0.0042 ± 0.00037	0.131 ± 0.050	F 1.44 (1, 379)<br>P 0.231
Weight at time of sacrifice (mg)			23.8 ± 5.36<br>**F 19.6 (1, 378)**<br>**P < 0.001**	F 1.07 (1,374)<br>P 0.302
Block (B2)<br>(B3)<br>(B4)	-0.000024 ± 0.00028<br>0.00104 ± 0.00036<br>0.000017 ± 0.00027<br>**F 3.67 (3, 328)**<br>**P 0.013**	-0.00013 ± 0.00018<br>0.00078 ± 0.00023<br>-0.000025 ± 0.00018<br>**F 5.62 (3, 320)**<br>**P 0.0009**	0.022 ± 0.0355<br>-0.034 ± 0.0434<br>-0.234 ± 0.0349<br>**F 21.1 (3, 378)**<br>**P < 0.001**	-13.81 ± 6.37<br>-21.6 ± 7.72<br>-39.1 ± 6.53<br>**F 12.4 (3, 382)**<br>**P < 0.001**

Symbols

Numbers within brackets – degrees of freedom

B – 50% cellulose diet

C – 70% cellulose diet

The intercept is the 30% cellulose treatment.
