# Supplementary material for: Within- and Trans-Generational Effects of Variation in Dietary Macronutrient Content on Life-History Traits in the Moth Plodia interpunctella
Source: PLoS One. 2016 Dec 29;11(12):e0168869. doi: 10.1371/journal.pone.0168869 (PMC5199116; doi:10.1371/journal.pone.0168869)
Supplement: S2 Table — Effects of dietary macronutrient composition on fecundity. (PDF) [file pone.0168869.s002.pdf]

Supporting information for "Within- and trans-generational effects of variation in dietary macronutrient content on life-history traits in the moth *Plodia interpunctella*"

Joanne E. Littlefair, Robert J. Knell

S2 Table: Full table of statistics for maternal fecundity. Effects of dietary macronutrient composition on fecundity.

	Fecundity
Intercept	111 ± 35.9
Interaction between nutrient content and P:C ratio	LR 1.90 (2)<br>P 0.387
Total nutrient content	LR 0.275 (2)<br>P 0.872
P:C ratio	LR 1.15 (1)<br>P 0.283
Maternal weight	8091 ± 2331<br>**LR 4.72 (1)**<br>**P 0.031**
Maternal adult lifespan	-17.5 ± 2.67<br>**LR 42.4 (1)**<br>**P < 0.001**
Random effects (block)<br>Stdev of intercept<br>Stdev of residual	 11.6<br>63.6

<u>Symbols</u>

LR – likelihood ratio test statistic

Numbers within brackets – degrees of freedom
