# Supplementary material for: Within- and Trans-Generational Effects of Variation in Dietary Macronutrient Content on Life-History Traits in the Moth Plodia interpunctella
Source: PLoS One. 2016 Dec 29;11(12):e0168869. doi: 10.1371/journal.pone.0168869 (PMC5199116; doi:10.1371/journal.pone.0168869)
Supplement: S3 Table — Transgenerational effects of maternal dietary macronutrient composition on offspring life history traits. (PDF) [file pone.0168869.s003.pdf]

Supporting information for: "Within- and trans-generational effects of variation in dietary macronutrient content on life-history traits in the moth *Plodia interpunctella*"

Joanne E. Littlefair, Robert J. Knell

S3 Table: Full table of statistics for transgenerational effects. Transgenerational effects of maternal dietary macronutrient composition on offspring life history traits.

Transgenerational effects	Pupation weight (g)	Eclosion weight (g)	Phenoloxidase vmax (square-root)	Total haemocyte count	Larval development time (days)	Adult lifespan (days)
Intercept	0.0145 ± 0.0002	0.0109 ± 0.0001	1.03 ± 0.132	117 ± 11.5		
Interaction between nutrient content and P:C ratio	χ 0.603<br>P 0.740	χ 0.717<br>P 0.699	χ 4.13 (2)<br>P 0.127	LR 1.10<br>P 0.576	χ 1.48 (2)<br>P 0.478	χ 1.15 (2)<br>0.562
Total nutrient content	χ 4.22<br>P 0.121	χ 3.81<br>P 0.149	χ 1.04 (2)<br>P 0.595	LR 0.372<br>P 0.830	χ 2.73 (2)<br>P 0.256	χ 2.17 (2)<br>P 0.338
P:C ratio	χ 0.013<br>P 0.910	χ 0.038<br>P 0.844	χ 0.245 (1)<br>P 0.621	LR 1.99<br>P 0.159	χ 1.95 (1)<br>P 0.163	χ 0.245 (1)<br>P 0.621
Maternal weight	χ 0.0227<br>P 0.880	χ 0.025<br>P 0.874	χ 0.221 (1)<br>P 0.638	LR 1.47<br>P 0.225		
Weight at time of larval sacrifice			-18.4 ± 5.17<br>**χ 12.1 (1)**<br>**P 0.0005**	-1240 ± 467<br>**LR 7.04**<br>**P 0.0086**		
Pupation weight						-227 ± 32.4<br>**χ 50.1 (1)**<br>**P < 0.001**
Random effects:<br>Block<br>Intercept<br><br>Family in block<br>Intercept<br>Residual	 <br>0.0000<br><br><br>0.00155<br>0.00251	 <br>0.0000<br><br><br>0.00099<br>0.00182	 <br>0.1220<br><br><br>0.2345<br>0.3543	 <br>8.61<br><br><br>0.00649<br>36.8	 <br>0.2252<br><br><br>0.8050	 <br>0.2175<br><br><br>0.2901

<u>Symbols</u>

X – chi squared test statistic

LR – likelihood ratio test statistic

Numbers within brackets – degrees of freedom
